# Shear-Stress-Dependent Viscous Properties of Hyaluronic-Based Lubricants

**DOI:** 10.3390/jcm14248753

**Published:** 2025-12-10

**Authors:** Ulrich Graf, Doreen Schmidl, Gerhard Garhöfer, Leopold Schmetterer

**Affiliations:** 1 Department of Clinical Pharmacology, Medical University of Vienna, 1090 Vienna, Austria; ulrich.graf@meduniwien.ac.at (U.G.); doreen.schmidl@meduniwien.ac.at (D.S.); gerhard.garhoefer@meduniwien.ac.at (G.G.); 2 Center for Medical Physics and Biomedical Engineering, Medical University of Vienna, 1090 Vienna, Austria; 3 Singapore Eye Research Institute, Singapore National Eye Centre, Singapore 169856, Singapore; 4 Ophthalmology and Visual Sciences Academic Clinical Program, Duke-NUS Medical School, Singapore 169857, Singapore; 5 SERI-NTU Advanced Ocular Engineering (STANCE), Nanyang Technological University, Singapore 639798, Singapore; 6 School of Chemistry, Chemical Engineering and Biotechnology, Nanyang Technological University, Singapore 637459, Singapore; 7 Fondation Ophtalmologique Adolphe De Rothschild, 75019 Paris, France; 8 Aier Eye Hospital Group, Changsha 410000, China

**Keywords:** dry-eye disease, ophthalmology, topical lubricants, shear stress

## Abstract

**Background/Objectives**: The physical properties of topical eyedrop formulations used for the treatment of dry-eye disease play an important role in the lubrication of the ocular surface. In the present study, we investigate the shear-stress-dependent viscous properties of seven different commercially available lubricants using a novel optical rheometer for accurate analysis of the viscosity of liquid samples. In addition, the viscosity of natural tears was studied. **Methods**: Viscosity measurements were performed using Fluidicam RHEO technology (FORMULACTION, Toulouse, France), an automated optical rheometer combining microfluidic and imaging technologies. Measurements were conducted at a temperature of 34 °C and at shear rates ranging from 3000 s^−1^ to 30,000 s^−1^ to mimic conditions during eye blinking. **Results**: Natural tears showed minimal change in viscosity in response to changes in shear stress, with viscosity values of 0.91 mPa·s at 3000 s^−1^ and 0.80 mPa·s at 30,000 s^−1^. Among the artificial tear formulations, Thealoz^®^ Duo had the lowest viscosity (2.62 ± 0.01 mPa·s at 3000 s^−1^), followed by Ivizia^®^ (2.42 ± 0.02 mPa·s), Hylo Comod^®^ (3.69 ± 0.01 mPa·s), Hylo Parin^®^ (3.87 ± 0.01 mPa·s), Xailin HA^®^ (4.73 ± 0.02 mPa·s), Vismed^®^ (5.42 ± 0.02 mPa·s), and Systane Hydration^®^ (7.76 ± 0.1 mPa·s). **Conclusions**: This study demonstrates that commercially available ocular lubricants exhibit varying degrees of shear-thinning behavior, a finding that is clinically relevant for their performance on the ocular surface. Formulations containing low-molecular-weight hyaluronic acid, such as Thealoz^®^ Duo, exhibited viscosity values closest to those of natural tears at the measured shear rates.

## 1. Introduction

Dry-eye disease (DED) is a multifactorial disease that impacts vision and patient-related quality of life. With a global prevalence of 5–50% of the population depending on geographic location and diagnostic criteria, DED represents a significant public health concern [1]. The condition has been increasing in prevalence due to aging populations, extended screen times, and environmental factors such as air pollution and low-humidity environments [2].

DED is characterized by tear film instability, hyperosmolarity, and inflammation of the ocular surface, resulting in symptoms ranging from discomfort and visual disturbances to potential damage to the ocular surface [3,4]. Management of DED typically begins with artificial tear supplements, which remain the cornerstone of therapy despite advances in anti-inflammatory treatments for more severe cases [5].

Natural tears exhibit complex rheological properties, including non-Newtonian, shear-thinning behavior, where viscosity decreases as shear rate increases [6]. This property is crucial for tear function—higher viscosity when the eye is open helps maintain tear-film stability, while lower viscosity during blinking reduces friction between the lid and ocular surface [7]. Thus, artificial tears should mimic the non-Newtonian properties of natural tears, providing adequate lubrication while maintaining optical clarity.

Hyaluronic acid (HA) has emerged as a prominent component in artificial tear formulations due to its favorable properties. As a naturally occurring glycosaminoglycan, HA possesses excellent water-binding capacity, biocompatibility, and viscoelastic characteristics [8]. This viscoelastic behavior—characterized by higher viscosity at low shear rates and reduced viscosity under dynamic conditions—closely resembles the non-Newtonian properties of natural tears and is essential for stabilizing the tear film during eye opening while minimizing mechanical friction during blinking [6,7].

The molecular weight of HA is a critical determinant of its rheological function [8]. In typical artificial tear formulations, hyaluronic acid serves as the primary viscosity agent at concentrations ranging from 0.10% to 0.40% [9], though concentrations up to 1.0% have been reported [10]. Generally speaking, higher-molecular-weight variants (>1000 kDa) typically confer greater viscosity and residence time but may impair spreadability or induce transient blurring, whereas lower-molecular-weight HA enhances ocular distribution but may reduce retention [11,12]. Commercial formulations often incorporate additional excipients such as trehalose, carboxymethyl cellulose, or lipids to improve biocompatibility and surface adhesion. However, the ratio of HA to co-polymer varies substantially across commercial products, with some formulations relying mainly on HA at higher concentrations, while others combine lower HA concentrations with complementary polymers to achieve target rheological properties [13]. Thus, overall rheological performance results from the complex interaction of HA’s molecular characteristics and those the other components of the formulation [5,8,14]. In summary, the rheological behavior of artificial tear formulations cannot be reliably predicted based solely on the molecular weight or concentration of hyaluronic acid, underscoring the need for empirical evaluation under physiologically relevant conditions [11,14].

In the past different technical approaches such as rotational rheometers, capillary viscometers, and microfluidic devices have been used to assess shear-thinning behavior of natural tears and artificial tear substitutes. However, each of these methods suffers from intrinsic limitations and differs in sensitivity to sample volume, evaporation and temperature control [6]. Thus, measuring shear thinning is technically challenging, and the interpretation of the results requires caution, because differences in instrument type, geometry, or measurement conditions can lead to substantial variation in reported rheological behavior, in particular if the measurements do not cover the entire shear-rate range relevant to physiological conditions [7,15,16].

Recent advances in microfluidic technology have enabled more precise rheological measurements across a broader range of shear rates, offering new insights into the physical properties of different artificial tear formulations [17]. Using this technology, in this study, we aimed to characterize the rheological properties of seven commercially available artificial tear formulations containing HA of various molecular weights, utilizing a microfluidic-based optical rheometer to measure viscosity at physiologically relevant temperature and shear rates. Natural tears were also evaluated for comparison, providing a benchmark for assessing how closely these formulations approximate the rheological properties of the natural tear film.

## 2. Materials and Methods

### 2.1. Compounds Investigated

The following 7 different artificial tear formulations were evaluated in this study: 2 artificial tear formulations containing high-molecular-weight hyaluronic acid (>1000 kDa), namely, Hylocomod^®^ and Hyloparin^®^ (both Ursapharm Arzneimittel GmbH, Saarbrücken, Germany); 2 formulations containing medium-molecular-weight (500–1000 kDA) hyaluronate, namely, Xailin HA^®^ (VISUfarma BV, Amsterdam, The Netherlands) and Vismed^®^ (Horus pharma, Nice, France); and 3 formulations with low-molecular-weight hyaluronate (<500 kDa), namely, Systane hydration^®^ (Alcon Inc., Geneva, Switzerland), Thealoz^®^ Duo (Laboratories Théa, Clermont-Ferrand, France), and IVIZIA^®^ (Théa Pharma Inc., Waltham, MA, USA). An overview of the compositions of the 7 commercially available artificial tears is provided in Table 1. Furthermore, a natural tear sample has been analyzed as a standard reference.

### 2.2. Measurement of Rheological Properties

Viscosity measurements were conducted using the commercially available Fluidicam RHEO system (FORMULACTION, Toulouse, France), a novel instrument that combines microfluidic technology with optical imaging analysis. Unlike conventional rotational rheometers that apply mechanical shear directly to samples, this system employs a co-flow microfluidic principle where measurements are derived from the relative positioning of fluid interfaces. Briefly, the measurement process involves simultaneous introduction of two fluids into a microfluidic channel: the sample (artificial tear) and a reference fluid with known viscosity. Both fluids flow side by side in a laminar regime through a rectangular microchannel (2.2 mm width) without mixing. The system’s camera captures the position of the interface between the two fluids; this position shifts based on the viscosity ratio and flow rate ratio according to the following relationship:

$$\frac{W}{W_{R}}=\frac{\eta }{\eta_{R}}\frac{Q}{Q_{R}}$$

where *W* and *W_R_* represent the width of the channel occupied by the sample and reference fluid, respectively; *η* and *η_R_* are the viscosities of the sample and reference fluid; and *Q* and *Q_R_* denote the volumetric flow rates of the sample and reference fluid.

During measurement, the system automatically adjusts the flow rates to maintain the interface at the center of the channel. Once equilibrium is established, the viscosity is calculated from the known parameters of the reference fluid and the applied flow rates as follows:

$$\dot{\gamma }=\frac{6\;Q}{h^{2}\;w}$$

where $\dot{\gamma }$ is the generated shear rate (s^−1^), *Q* is the volumetric flow rate generated by the sample syringe pumps (µL/sec), h represents the channel height in µm, and *W* is the channel width occupied by the sample in mm.

Two microfluidic chips with different channel heights (150 µm and 50 µm) were employed to cover two different ranges of shear rates. The 150 µm chip allowed for measurements in the range of 100–18,000 s^−1^, while the 50 µm chip enabled measurements at higher shear rates (1000–180,000 s^−1^).

All viscosity measurements were conducted at 34 °C to simulate ocular surface temperature. For each measurement point, the 10 measurements were made and averaged to ensure reproducibility and minimize experimental error. Flow curves were generated by progressively increasing shear rates and recording the corresponding viscosity values. Particular attention was paid to measurements at a shear rate of 3000 s^−1^, which represents the physiological range of shear rates experienced on the ocular surface during normal blinking.

### 2.3. Statistical Analyses

Descriptive analysis was performed using GraphPad Prism version 7.00 (GraphPad Software, La Jolla, CA, USA). Repeated-measures ANOVA was used to assess differences between flow curves. A *p* < 0.05 was considered statistically different. All results are presented as the mean ± standard error of the mean (SEM).

## 3. Results

**Figure 1:**
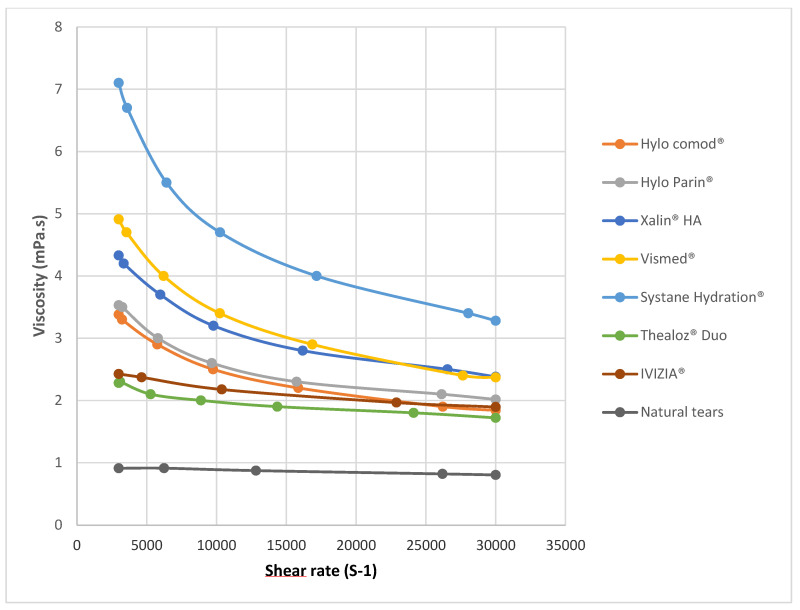
Rheological behavior of commercial artificial tear formulations containing high-molecular-weight (>1000 kDa), medium-molecular-weight (500 kDa to 1000 kDa), and low-molecular-weight (<500 kDa) sodium hyaluronate.

Analysis of the rheological profiles revealed distinct shear-thinning behavior across all the ocular lubricants tested, with notable differences in magnitude and pattern based on formulation composition (Figure 1). The viscosity of natural tears at a shear rate of 3000 s^−1^ was determined to be 0.91 ± 0.02 mPa·s, serving as the physiological reference point for comparing commercial formulations. The viscosity over the range of shear rates was significantly different for natural tears relative to that of all the other lubricants tested (*p* < 0.05).

At this shear rate, Ivizia^®^ and Thealoz^®^ Duo exhibited the lowest viscosity among the tested products—2.43 ± 0.02 mPa·s and 2.62 ± 0.01 mPa·s, respectively. The formulations containing high-molecular-weight hyaluronic acid, Hylo Comod^®^ and Hylo Parin^®^, showed moderately higher viscosities—3.69 ± 0.01 mPa·s and 3.87 ± 0.01 mPa·s, respectively. For the formulations containing medium-molecular-weight hyaluronic acid, Xailin HA^®^ and Vismed^®^, measurements indicated viscosities of 4.73 ± 0.02 mPa·s and 5.42 ± 0.02 mPa·s, respectively, while Systane Hydration^®^ demonstrated the highest viscosity, 7.76 ± 0.1 mPa·s, at a shear rate of 3000 s^−1^.

**Figure 2:**
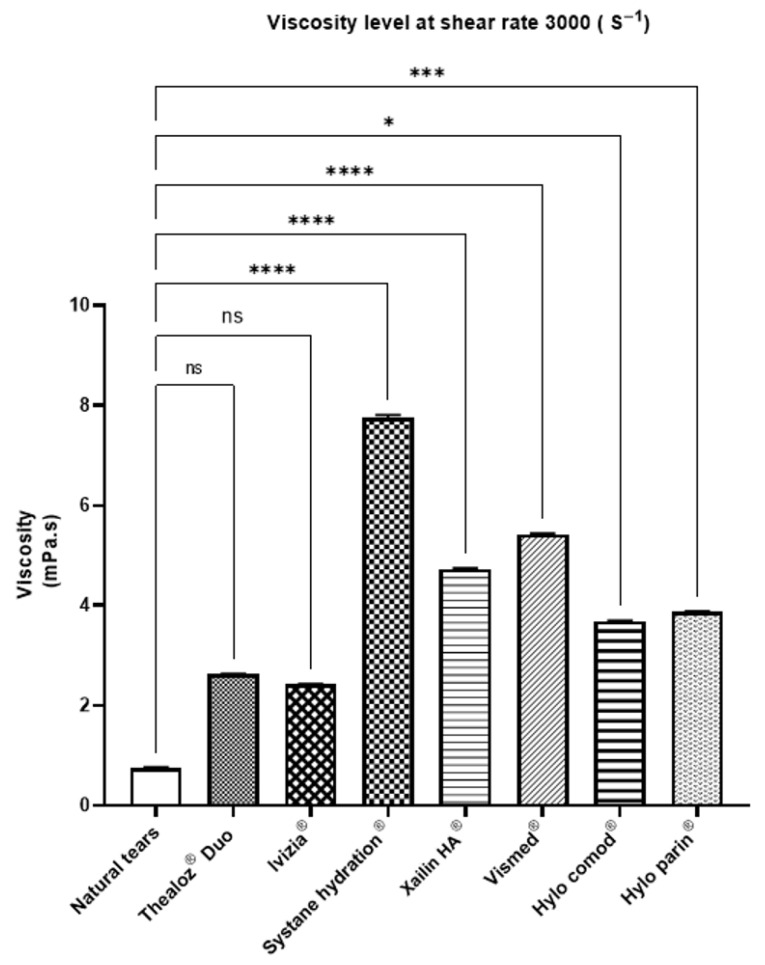
Viscosity at a shear rate of 3000 s^−1^ of 8 commercially artificial tear formulations measured using the Fluidicam system. HA denotes hyaluronate. Significance levels: * *p* < 0.05, *** *p* < 0.001, **** *p* < 0.0001, ns = not significant.

The shape of the rheological curves varied considerably across products (Figure 1 and Figure 2). When examining the entire rheological curves, all formulations maintained their non-Newtonian characteristics throughout the tested range. Formulations containing medium-molecular-weight hyaluronic acid (Xailin HA^®^ and Vismed^®^) and high-molecular-weight hyaluronic acid (Hylo Comod^®^ and Hylo Parin^®^) exhibited steep viscosity reductions as shear rates increased. In contrast, formulations with low-molecular-weight hyaluronic acid demonstrated more varied behavior. Thealoz^®^ Duo displayed a more moderate shear-thinning pattern with a more linear viscosity compared to the formulations with high- and medium-molecular-weight. Systane Hydration^®^, despite its lower-molecular-weight-hyaluronic-acid content, demonstrated the most significant shear-thinning behavior of all the tested products at low shear rates.

## 4. Discussion

The rheological behavior of artificial tear formulations plays a central role in their clinical performance, particularly in mild to moderate forms of dry-eye disease (DED). Natural tears exhibit non-Newtonian, shear-thinning behavior—meaning their viscosity decreases as the shear rate increases. This adaptive property is physiologically significant: during periods of eye opening, elevated viscosity stabilizes the tear film and prevents evaporation, while during blinking, lower viscosity minimizes friction between the eyelids and ocular surface, reducing mechanical stress. Thus, it does not come as a surprise that many of the commercially available products are designed to mimic the viscoelastic properties of the tear film. In this study, we assessed the rheological profiles of seven commercially available artificial tears as well as tear fluid using an optical microfluidic rheometer, which enabled high-precision viscosity measurements over a broad range of physiologically relevant shear rates.

Among the tested formulations, Ivizia^®^ exhibited the lowest viscosity at a shear rate of 3000 s^−1^, followed by Thealoz^®^ Duo. Although the active ingredient of Ivizia^®^ is povidone, both of these formulations contain low-molecular-weight hyaluronic acid (0.15%) combined with trehalose, and they demonstrated viscosity profiles most similar to natural tears. While these rheological similarities to natural tears are promising, it remains unclear to what extent these in vitro findings translate into clinical benefits in routine practice. However, the combination of trehalose and hyaluronic acid has shown promising clinical outcomes, including significant improvements in tear film thickness, symptom relief, and ocular surface protection across multiple studies conducted on patients with moderate to severe dry eye disease, supporting the effectiveness of this combination [18,19,20]. Moreover, a recent post hoc analysis of three clinical trials confirmed that artificial tears containing trehalose and low-molecular-weight hyaluronic acid significantly improved OSDI scores, tear break-up time, and ocular staining [21]. Miyata et al. found that low-molecular-weight hyaluronate is more effective than high-molecular-weight hyaluronate at protecting corneal endothelial cells and the cornea during surgery, again supporting the clinical efficacy of low-molecular-weight agents [21,22].

Interestingly, the high-molecular-weight formulations Hylo Comod^®^ and Hylo Parin^®^ showed moderately higher viscosities than the formulations with low-molecular-weight hyaluronic acid, despite their lower hyaluronic acid concentration (0.10%), whereas the formulations with medium-molecular-weight HA such as Xailin HA^®^ and Vismed^®^ were among the highest viscous agents in the study. These findings highlight that viscosity is not solely determined by the concentration or molecular weight of hyaluronic acid; it is also influenced by the overall formulation matrix, including copolymers and osmoprotectants [14]. These findings also align with previous data. Aragona et al. observed that HA concentration often exerts greater influence on viscosity than molecular weight alone, particularly at concentrations above 0.15–0.18% [14]. In their study, formulations with HA concentrations of 0.18–0.24% generally exhibited higher viscosities than those with 0.10–0.15%, even when the latter contained higher-molecular-weight HA [14]. Similarly, Kapadia et al. reported that the presence of co-formulants can dramatically alter rheological profiles, with synergistic effects occurring between HA and agents like hydroxypropyl guar or carboxymethylcellulose [11]. This is also reflected in our data indicating that Systane Hydration^®^, which contains hydroxypropyl guar in addition to low-molecular-weight HA, displayed the highest viscosity among all products tested, despite its low HA weight.

Previous studies have reported viscosity values for natural tears of about 0.65 mPa·s, which is considerably higher than the 0.91 ± 0.02 mPa·s measured in the current study [6]. Although the exact reason for this discrepancy is not entirely clear, it has to be noted that direct comparison with previously reported values is complicated by several factors. First and most importantly, the shear viscosity of ocular lubricants is highly temperature-dependent. As such, a mean decrease of approximately 21% in shear viscosity was reported for 12 commonly used formulations when the temperature increased from room temperature (24.5 °C) to corneal surface temperature (34.5 °C) [6]. In the current study, rheological measurements were performed at 34 °C, whereas the majority of earlier studies were conducted at room temperature rather than under physiologically relevant conditions [7,23]. This may at least partially contribute to the observed differences. Second, natural tears exhibit considerable inter-individual variability in composition—including in terms of protein, lipid, mucin, and electrolyte content—which may significantly influence measured viscosity [24,25]. Third, differences in measurement techniques may have contributed to the differing results. We used an optical microfluidic rheometer, which operates on fundamentally different principles compared to conventional rotational rheometers used in previous studies [7,16]. Further studies are needed to fully characterize natural tear viscosity with different complementary measurement techniques. Additionally, establishing a standardized protocol for tear collection, handling, and measurement conditions may reduce variability and would advance our understanding of tear film rheology in health and disease.

The shape of the rheological curves varied considerably across the tested products (Figure 1). When examining the rheological profiles, all formulations maintained their non-Newtonian characteristics throughout the tested range, though with notable differences in the gradient of shear-thinning behavior. Formulations containing medium-molecular-weight (Xailin HA^®^ and Vismed^®^) and high-molecular-weight hyaluronic acid (Hylo Comod^®^ and Hylo Parin^®^) exhibited steep viscosity reductions as shear rates increased. This pronounced decrease most probably reflects substantial structural reorganization within the polymer network under increasing shear forces, a finding that aligns with previous rheological analyses of solutions with high-molecular-weight hyaluronic acid [8].

In contrast, formulations containing low-molecular-weight hyaluronic acid demonstrated more varied behavior patterns. Thealoz^®^ Duo displayed a more moderate shear-thinning pattern with a more linear viscosity profile compared to high- and medium-molecular-weight products. This suggests a more gradual reconfiguration of its molecular structure under increasing shear forces. Systane Hydration^®^, despite its lower-molecular-weight-hyaluronic-acid content, demonstrated the most significant shear-thinning behavior of all the tested products at low shear rates. This unexpected finding may stem from the synergistic effects between hyaluronic acid and hydroxypropyl guar within this formulation, as previously documented by Aragona et al. [14].

There are some limitations of the current study that need to be considered. First, there is no universally accepted consensus on the exact shear rates that reflect clinical conditions on the ocular surface. Physiological estimates suggest that ocular shear rates can be as low as 0.03 s^−1^ during blepharospasm and may rise to between 4250 and 28,000 s^−1^ during blinking [6,11]. However, these values can vary substantially depending on individual blinking behavior, activity levels, and fatigue. To account for this variability and better simulate real-world conditions, we conducted measurements across a wide range of shear rates, enabling a comprehensive evaluation of how each artificial tear formulation behaves under both resting and dynamic ocular conditions [15]. Among these rates, a shear rate of 3000 s^−1^ was chosen as a representative benchmark in this study. Although previous work has examined the full spectrum of shear rates from 0 to approximately 30,000 s^−1^ [15], our approach accurately simulates blink dynamics but does not capture low-shear behavior. Thus, in future studies, expanding the shear rate range to include low shear conditions would provide additional valuable insights into formulation behavior during prolonged inter-blink periods.

Secondly, a limitation of this study lies in the incomplete compositional transparency of the tested formulations. Although we included artificial tears containing hyaluronic acid of reportedly different molecular weights, manufacturers rarely disclose the exact molecular weight values, instead categorizing this compound broadly as “high,” “medium,” or “low” molecular weight. This lack of transparency complicates direct comparisons between products and limits the ability to establish clear correlations between molecular weight and rheological behavior.

One of the strengths of the current study is that we employed a microfluidic-based optical rheometer for rheological characterization rather than a conventional rotational rheometer. This methodological approach offers several significant advantages for the assessment of ocular lubricants. The optical rheometer does not require calibration or zero-gap setting procedures that are mandatory in standard rotational rheometers, thus eliminating potential sources of measurement error [26]. The microfluidic system demonstrates exceptional sensitivity for detecting minute viscosity variations across an extensive shear rate range (100–180,000 s^−1^) utilizing only two microfluidic chip configurations [17]. Furthermore, the confined nature of the microfluidic chamber renders it particularly advantageous for analyzing volatiles, an aspect that is especially relevant for ocular-surface lubricants. In future studies, researchers can perform comprehensive clinical trials directly correlating in vitro rheological profiles with clinical outcomes. This would allow clear determination of which viscosity profiles translate into superior symptom relief, improved tear-film stability, and enhanced patient satisfaction.

In summary, our rheological analysis revealed distinct shear-thinning behaviors across all seven commercial artificial tear formulations and natural tears, with Ivizia^®^ and Thealoz^®^ Duo exhibiting profiles most similar to the profile of natural tears. Medium- and high-molecular-weight formulations demonstrated steeper viscosity gradients, while our findings confirmed that rheological behavior depends not solely on hyaluronic acid concentration or molecular weight but on the complex interplay with co-polymers and additional excipients.

## Figures and Tables

**Table 1:** Contents of 7 commercially available artificial tears.

Artificial Tear *	SH (%)	Other Active Ingredients	Other Ingredients
Hylo Comod^®^	0.10	-	citrate buffer, sorbitol
Hylo Parin^®^	0.10	heparin	glycerol, CA, sodium citrate
Xailin HA^®^	0.20	-	SP
Vismed^®^	0.18	-	-
Systane Hydration^®^	0.15	HG	AMP, BA, PEG, PG
Thealoz^®^ Duo	0.15	trehalose	-
Ivizia^®^	0.15	Povidone (0.5%)	trehalose

* All product names are (^®^) registered trademarks; SH denotes sodium hyaluronate; PEG denotes polyethylene glycol; PG denotes propylene glycol; HG denotes hydroxypropyl guar; AMP denotes aminomethylpropanol; BA denotes boric acid; SP denotes sodium perborate; CA denotes citric acid.

## Data Availability

The data presented in this study are available on request from the corresponding author due to confidentiality restrictions.

## Abbreviations

The following abbreviations are used in this manuscript:

AMP	Aminomethylpropanol
BA	Boric acid
CA	Citric acid
DED	Dry-eye disease
HA	Hyaluronate
HG	Hydroxypropyl guar
PEG	Polyethylene glycol
PG	Propylene glycol
SEM	Standard error of the mean
SH	Sodium hyaluronate
SP	Sodium perborate
